# Motor cortex plasticity response to acute cardiorespiratory exercise and intermittent theta-burst stimulation is attenuated in premanifest and early Huntington’s disease

**DOI:** 10.1038/s41598-021-04378-2

**Published:** 2022-01-20

**Authors:** Sophie C. Andrews, Dylan Curtin, James P. Coxon, Julie C. Stout

**Affiliations:** 1 School of Psychological Sciences and Turner Institute for Brain and Mental Health, Monash University, Clayton, VIC Australia; 2 Neuroscience Research Australia, 139 Barker Street, Randwick, NSW Australia; 3 School of Psychology, University of New South Wales, Sydney, NSW Australia

**Keywords:** Neuroscience, Neurology

## Abstract

Huntington’s disease (HD) mouse models suggest that cardiovascular exercise may enhance neuroplasticity and delay disease signs, however, the effects of exercise on neuroplasticity in people with HD are unknown. Using a repeated-measures experimental design, we compared the effects of a single bout of high-intensity exercise, moderate-intensity exercise, or rest, on motor cortex synaptic plasticity in 14 HD CAG-expanded participants (9 premanifest and 5 early manifest) and 20 CAG-healthy control participants, using transcranial magnetic stimulation. Measures of cortico-motor excitability, short-interval intracortical inhibition and intracortical facilitation were obtained before and after a 20-min bout of either high-intensity interval exercise, moderate-intensity continuous exercise, or rest, and again after intermittent theta burst stimulation (iTBS). HD participants showed less inhibition at baseline compared to controls. Whereas the control group showed increased excitability and facilitation following high-intensity exercise and iTBS, the HD group showed no differences in neuroplasticity responses following either exercise intensity or rest, with follow-up Bayesian analyses providing consistent evidence that these effects were absent in the HD group. These findings indicate that exercise-induced synaptic plasticity mechanisms in response to acute exercise may be attenuated in HD, and demonstrate the need for future research to further investigate exercise and plasticity mechanisms in people with HD.

## Introduction

Huntington’s disease (HD) is an autosomal dominant neurodegenerative disease resulting in motor dysfunction, cognitive impairment and neuropsychiatric symptoms^1^. Onset is typically in midlife, although the onset and course of HD is variable, which is proposed to be due to genetic and environmental factors, including lifestyle^2^. Exercise has emerged as a promising lifestyle candidate to modify disease onset and progression^2^. Research using HD mouse-models demonstrates that cardiovascular exercise alters biomarkers of neuroplasticity, such as brain derived neurotrophic factor (BDNF)^3,4^, and delays signs of disease^5–8^. Additionally, one retrospective study of people with HD found that a lifestyle comprising less time sitting was associated with later disease onset^9^. Recently, a number of exercise interventions have been trialed in HD with mixed results^10–13^, although one study provided preliminary evidence that a 9 month multidisciplinary intervention that included exercise increased grey matter volume and improved learning and memory^13^. One of the key obstacles to designing effective exercise interventions in HD is the lack of clarity regarding the neurophysiological mechanisms that underlie the effect of exercise on the brain in people with the HD CAG-expansion.

A strong candidate mechanism is that each bout of exercise opens a window wherein the capacity for synaptic plasticity is enhanced^14^. Synaptic plasticity refers to the biological process that modifies the strength of communication between neurons^15^. In healthy adults, an acute bout of cardiovascular exercise can transiently increase synaptic plasticity in the motor cortex^16^. For example, 20–30 min of moderate-intensity continuous cycling reduces inhibition^17,18^, increases facilitation^19^, and amplifies the response to a plasticity induction protocol^20^. Additionally, high-intensity interval training (HIIT), where short intervals of high-intensity exercise are interspersed with periods of less intense exercise, elevates BDNF levels^21^, which may further benefit synaptic plasticity^21–23^. Recently, we reported the first direct comparison of high-intensity interval exercise and moderate-intensity continuous exercise on synaptic plasticity in motor cortex. We induced a long-term potentiation (LTP) ‘like’ effect by using intermittent theta burst stimulation (iTBS), which comprises brief, high-frequency subthreshold trains of cortical magnetic stimulation pulses in the gamma frequency band (50 Hz), superimposed upon a theta rhythm (5 Hz)^24,25^. In a sample of 20 healthy adults, we showed that high-intensity interval exercise combined with iTBS enhanced synaptic plasticity, compared to rest, with moderate-intensity continuous exercise showing an intermediate response^26^. Whether high-intensity exercise is optimal to enhance synaptic plasticity in people living with neurodegenerative disease is currently unknown.

To date, no study has investigated the acute effects of exercise on motor cortex plasticity in HD. This is important because there is evidence that early disruption to cortico-striatal circuits reduces inhibition in the motor cortex^27^, and previous studies have shown that people with the CAG-expansion for HD have attenuated plasticity responses to several non-invasive brain stimulation protocols^28–31^. The absence of the normal neuroplasticity response may be attributable to altered or dysregulated dopamine signaling, as well as reduced production of BDNF in HD. Specifically, the indirect (D2) dopaminergic pathway within the striatum is affected early in HD. The indirect pathway connects to the motor cortex via the nigrostriatal pathway^32^, and is important for motor cortex plasticity^27^. Additionally, mutant huntingtin has been found to decrease the level of BDNF and its receptor tropomyosin-related kinase B (TrkB) in human and mouse brains, and reduced release of BDNF has been observed in cortical neurons of an HD mouse-model^33^. Given its known effects on dopamine and BDNF, exercise could present a possible early intervention strategy for people with the HD CAG-expansion to counteract these neurophysiological changes and boost neuroplasticity, optimize cognitive reserve, and delay symptom onset or slow progression^34^. The aim of the current study was therefore to investigate whether a single bout of either HIIT or moderate-intensity continuous exercise enhances the response to iTBS in people with premanifest and early manifest HD, in comparison to an HD-CAG healthy control group. Using our established transcranial magnetic stimulation (TMS) methodology^26^, we measured how exercise intensity alters inhibition and excitation in the motor cortex, and synaptic plasticity as induced by iTBS. We hypothesized that similar to healthy controls, HD gene-expanded participants would show the largest enhancement of synaptic plasticity following high-intensity, rather than moderate-intensity exercise (compared to rest). An alternative hypothesis was that baseline alterations to motor cortex neurophysiology (e.g., reduced inhibition and increased facilitation) would attenuate this effect.

## Methods

### Participants

The sample comprised 20 CAG healthy control participants (Control Group), and 14 HD CAG gene-expanded participants (HD Group; 9 premanifest, 5 early manifest) recruited from the ENRU-Stout HD database held at Monash University, and the Statewide Progressive Neurological Disease Service at Calvary Health Care Bethlehem in Melbourne, Australia. Participants were aged between 21 and 70 years, right-handed, and were screened for contraindications to TMS^35^ and exercise (e.g., cardiovascular disease, uncontrolled hypertension)^36^. Exclusion criteria were a history of any psychiatric or neurological illness (except HD for the HD group), seizure, any serious medical conditions, or current pregnancy.

For the HD Group, participants were required to have a genetically-confirmed expansion of the HD CAG repeat sequence (≥ 39 CAG repeats), and no more than mild functional impairment, defined as a Total Functional Capacity (TFC) score of ≥ 11^37^ on the Unified Huntington’s Disease Rating Scale^38^ (UHDRS). Participants were classified as premanifest if they had never received a clinical diagnosis of HD, and as early HD if they had received a clinical diagnosis of HD. Disease Burden Score (calculated as age × [CAG-35.5]) ranged from 91 to 441^39^, equating to estimated years to onset ranging from 0 to 43 years. For those who also participated in the longitudinal, observational Enroll-HD study (n = 13), UHDRS Total Motor Score was obtained from their most recent annual visit. Handedness was self-reported using the Edinburgh Handedness Inventory^40^, anxiety and depression symptoms were measured using the Hospital Anxiety and Depression Scale^41,42^, and current physical activity levels were assessed using the International Physical Activity Questionnaire (IPAQ)^43^. Groups did not differ in age, gender distribution, years of education, self-reported anxiety or depression, or current levels of physical activity (see Table 1). Of the HD participants, three reported mild anxiety symptoms, one reported moderate anxiety symptoms, and two reported mild depression symptoms. Of the control participants, three reported mild anxiety symptoms, one reported moderate anxiety symptoms, and one reported mild depression symptoms. These rates were consistent with normative data for the HADS for the general adult population^44^. The study was approved by the Human Research Ethics Committees of Monash University (Project Reference: 2019-7055-37585) and Calvary Health Care Bethlehem (Project Reference: 16081804) in Melbourne, Australia, and all participants gave written informed consent. All procedures were performed in accordance with the World Medical Association Declaration of Helsinki.

**Table 1:** Participant characteristics.

	HD gene-expanded	Healthy controls	Test statistic	*p* value
N	14	20		
Age (years)	39.71 (13.73), 26–70	35.15 (13.25), 21–64	*T*_32_ = − .97	.34
Women	10 (71%)	12 (60%)	*χ*^2^ _1_ = .47	.49
Years of education	15.86 (2.31)	16.85 (3.05)	*T*_32_ = 1.03	.31
CAG repeat length	41.71 (1.82), 39–46	–		
Disease-burden score	246 (104), 91–441	–		
UHDRS-TMS (*n* = 13)	4.92 (8.98), 0–25	–		
UHDRS-TFC	12.46 (0.87), 11–13	–		
HADS anxiety	5.57 (3.39), 0–11	4.90 (3.34), 0–13	*T*_32_ = − .57	.57
HADS depression	3.42 (3.18), 0–10	2.65 (2.30), 0–8	*T*_32_ = − .83	.41
Handedness, EHI	+ 79.62 (15.13)	+ 80.20 (13.27)	*T*_*31*_ = .15	.88
BMI	24.52 (2.41)	23.73 (4.10)	*T*_32_ = − .64	.52
IPAQ	6949 (4916)	4681 (2287)	*T*_15.42_ = − 1.56	.14
Resting heart rate, BPM	63 (7.38)	68 (12.51)	*T*_31.28_ = 1.43	.16
Rest threshold (%MSO)	61 (10.96)	66 (10.26)	*F*_1,32_ = 1.65	.21
Active threshold (%MSO)	49 (8.23)	52 (9.28)	*F*_1,32_ = 1.28	.26
Test stimulus (%MSO)	78 (15.55)	79 (12.40)	*F*_1,32_ = .03	.86
Conditioning stimulus (%MSO)	41 (7.65)	41 (8.18)	*F*_1,32_ = .02	.90
iTBS stimulus (%MSO)	29 (5.09)	31 (4.97)	*F*_1,32_ = .69	.41
Baseline 1 mV (NC)	.95 (.25)	1.07 (.28)	*F*_1,32_ = 1.70	.20
Baseline SICI (C/NC ratio)	**.61 (.24)**	**.50 (.07)**	***F***_**1,32**_ **= 5.11**	**.03**
Baseline ICF (C/NC ratio)	1.19 (.33)	1.18 (.40)	*F*_1,32_ = .005	.94

Data are mean (SD), range or number (%).

Significant values are in bold.

*UHDRS-TMS* total motor score: possible scores range from 0 to 124, *UHDRS-TFC* total functional capacity: possible scores range from 0 to 13, *UHDRS-TMS* total motor score: possible scores range from 0 to 124, *HADS* hospital anxiety and depression scale, *EHI* Edinburgh handedness inventory, [range: − 100 (left-handed) to + 100 (right-handed)], *BMI* body mass index, *IPAQ* International physical activity questionnaire (higher scores indicate higher levels of physical activity), *BPM* beats per minute, *RMT* resting motor threshold, *MSO* maximum stimulator output, *AMT* active motor threshold with monophasic stimulation, *TS* test stimulus, *CS* conditioning stimulus, *iTBS* intermittent theta burst stimulation, *NC* non-conditioned, *C* conditioned, *SICI* short interval intracortical inhibition, *ICF* intracortical facilitation.

### Study design and methods

Each participant (HD, controls) completed three sessions separated by at least 72 h, with session order counterbalanced within each group, in a pseudorandom manner, to ensure there were no systematic differences in session order between groups. Participants were instructed to refrain from moderate and vigorous physical activity for 24 h prior to each session.

### Session protocol

**Figure 1:**
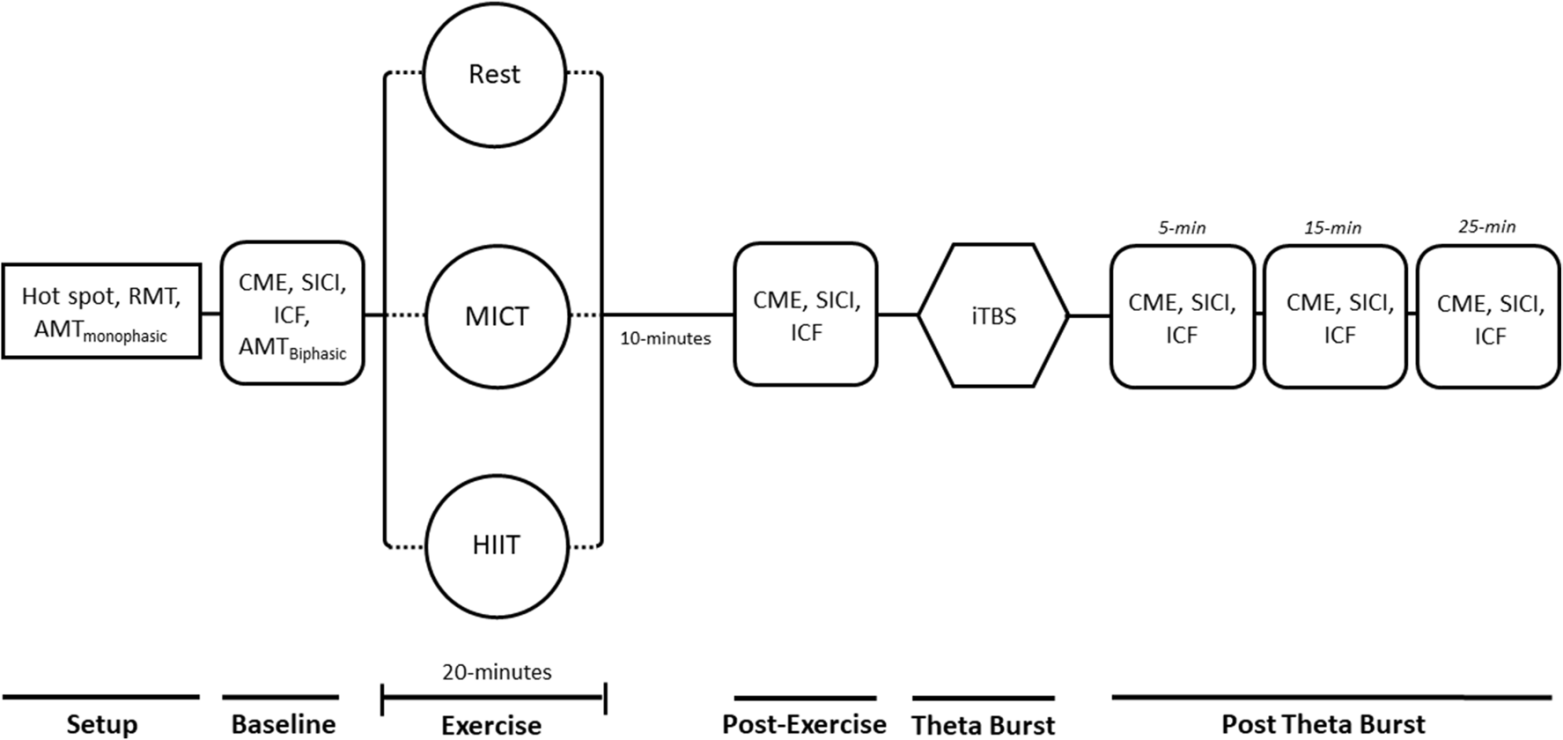
Sequence of events for each session. *RMT* resting motor threshold, *AMT *active motor threshold, *CME* cortico-motor excitability, *SICI* short-interval intracortical inhibition, *ICF* intracortical facilitation, *HIIT* high-intensity interval training, *MICT *moderate-intensity continuous training, *iTBS* intermittent theta burst stimulation. Participants completed one 20-min bout of exercise, or equivalent period of rest, per session.

Participants were fitted with a chest-strap heart-rate monitor (Polar H7, Polar Electro), then seated for the Baseline TMS assessment. Next, they completed 20 min of either high-intensity interval stationary cycling, moderate-intensity continuous stationary cycling, or rest, followed by a 10-min cool down, the post-exercise TMS assessment, iTBS, then Post-iTBS TMS assessments at 5, 15 and 25 min (see Fig. 1).

### TMS

We recorded several common single- and paired-pulse TMS measures, including corticomotor excitability (CME), short-interval intracortical inhibition (SICI), and intracortical facilitation (ICF)^26,45^. SICI is a measure of gamma-amino butyric acid (GABA) receptor A inhibition, whereas ICF has been linked to both the excitatory neurotransmitter glutamate and catecholamine function^46^. Obtaining both SICI and ICF enables assessment of the excitation-inhibition balance^26,47^.

The TMS protocol used in this study has been described elsewhere^26^. Briefly, participants were seated with both hands resting on a pillow on their laps. Surface electromyography (EMG) was recorded from first dorsal interosseous (FDI) of the right hand in a belly-tendon montage. Monophasic TMS pulses were applied to left primary motor cortex using a 70 mm diameter figure-of-eight coil connected to a MagVenture MagPro X100 stimulator (MagVenture Ltd.). At the beginning of each session we determined resting motor threshold, active motor threshold (i.e., the lowest stimulation intensity required to elicit MEPs of ≥ 200 μV during a tonic contraction of the FDI in at least 5 out of 10 consecutive trials^48^), and the test stimulus intensity required to produce a stable MEP of ~ 1 mV. Paired-pulse TMS was used to assess SICI and ICF, with the sub-threshold conditioning stimulus preceding the supra-threshold ~ 1 mV test stimulus by 2 ms and 12 ms, respectively^49^. The conditioning stimulus intensity was titrated to produce 50% inhibition of the non-conditioned (NC) MEP then held constant for all SICI and ICF measures. To ensure valid comparison of paired-pulse measures across different time-points, where necessary we adjusted the test stimulus, so that test MEP sizes were matched to within 30% of the comparison time-point (i.e. Post-Exercise was matched to Baseline, Post-iTBS was matched to Post-Exercise)^22,26^. For all single- and paired-pulse measurements, 16 stimuli were delivered at 5 s intervals.

iTBS was applied to the same motor cortex site, using the same TMS coil and stimulator, following standard procedure^24^. The iTBS intensity was set at 80% of biphasic active threshold, and three high frequency (50 Hz) biphasic pulses were delivered every 200 ms for 2 s, repeated every 10 s for 20 repetitions.

### Exercise

Participants completed the exercise sessions on a stationary cycle ergometer (Wattbike). To avoid stimulation of the FDI muscle during exercise sessions but not rest sessions, which could affect subsequent MEP size, we asked participants to refrain from gripping the handlebar of the bike during exercise. Heart rate was continuously monitored, and the intensity of each exercise session was tailored to individual participants based on their estimated heart rate reserve (HRR), which is an indication of the functional range of the heart during exercise. Resting heart rate was measured while participants were seated and HRR was computed by subtracting resting heart rate from age-predicted maximum heart rate (220—current age).

The high-intensity exercise protocol comprised alternating 3 min bouts of cycling at 50% HRR, with 2 min bouts of cycling at up to 90% HRR, for a total duration of 20 min^26^. The moderate-intensity exercise protocol comprised 20 min cycling at 50% HRR. For the rest session, participants sat quietly for 20 min. Participants self-reported their perceived exertion (RPE) using Borg’s scale (ranging from 6, no exertion at all, to 20, maximal exertion)^50^. Participants completed a 2-min warm-up and 2-min cool-down and the beginning and end of each exercise session, followed by a 10-min quiet rest period before the TMS protocol resumed.

With respect to the effectiveness of our methodology in manipulating exercise intensity methodology, we validated the high-intensity and moderate-intensity protocols by characterizing the effects of these sessions on percentage of HRR reached during each session, as well as power:weight ratio, cadence, and RPE, in each group (see Table 2). During the final epoch of the high-intensity exercise session, the HD and control groups reached a maximum of 93% and 91% HRR, respectively. In contrast, for the moderate intensity protocol, on average the HD and control groups exercised at 52% and 50% of HRR. During the high-intensity session, there were no differences between the groups on power, power:weight ratio, cadence, HRR, or RPE for any of the epochs (all *ps* > 0.08). During the moderate-intensity session, there were no differences between groups for power, power:weight ratio, cadence or HRR (all *ps* > 0.14;); however, the control group reported a higher perceived exertion than the HD group on average during the moderate intensity session *t*_28.7_ = 2.99, *p* = 0.006).

**Table 2:** Effects of high-intensity interval training and moderate-intensity continuous training on exercise performance.

Session	Power (Watts)		Power:Weight (Watts:kg)		Cadence (RPM)		%HRR		RPE	
	Controls	HD	Controls	HD	Controls	HD	Controls	HD	Controls	HD
**HIIT**										
0–3 min	68 (17.20)	68 (24.45)	1.00 (0.24)	1.05 (0.31)	76 (7.93)	73 (11.92)	43% (0.05)	45% (0.07)	10 (1.47)	10 (1.73)
4–5 min	106 (34.69)	105 (31.64)	1.56 (0.45)	1.61 (0.33)	90 (8.96)	88 (13.34)	62% (0.07)	64% (0.12)	13 (1.43)	13 (2.05)
6–8 min	71 (17.88)	75 (30.04)	1.05 (0.26)	1.16 (0.39)	77 (8.05)	75 (12.62)	52% (0.05)	52% (0.06)	11 (1.30)	11 (1.36)
9–10 min	111 (32.37)	114 (36.82)	1.63 (0.44)	1.73 (0.41)	95 (7.88)	90 (15.47)	73% (0.04)	74% (0.11)	13 (1.66)	13 (2.01)
11–13 min	73 (18.62)	76 (30.07)	1.07 (0.25)	1.17 (0.37)	78 (8.26)	75 (12.78)	57% (0.04)	58% (0.07)	11 (1.34)	11 (1.05)
14–15 min	122 (32.18)	126 (47.61)	1.80 (0.47)	1.90 (0.54)	101 (17.87)	96 (14.92)	82% (0.04)	80% (0.10)	14 (1.63)	15 (1.51)
16–18 min	72 (17.83)	73 (29.05)	1.06 (0.24)	1.11 (0.37)	77 (9.86)	75 (13.50)	62% (0.06)	59% (0.07)	12 (1.60)	12 (1.48)
19–20 min	156 (61.45)	165 (59.30)	2.30 (0.86)	2.51 (0.63)	105 (12.41)	108 (15.87)	91% (0.06)	93% (0.15)	18 (1.32)	17 (1.63)
**MICT**										
0–20 min	75 (19.14)	76 (27.39)	1.09 (0.23)	1.14 (0.37)	79 (9.78)	77 (10.87)	50% (0.05)	52% (0.03)	12 (1.18)	11 (0.54)

Data are mean (SD).

*HIIT* high-intensity interval training, *MICT* moderate-intensity continuous training, *RPM* revolutions per minute, *%HRR* percentage of heart rate reserve, *RPE* rating of perceived exertion.

### Data analysis

EMG recordings were inspected offline, and trials discarded if contaminated with muscle activity in the 100 ms before the TMS pulse, or if root mean square EMG was ≥ 10 μV, because muscle activity at the time of the TMS pulse is known to influence MEP amplitudes. For CME, MEP amplitudes were normalized to the mean MEP at the post-exercise time-point (i.e., a value of 1 was assigned to the post-exercise time-point and all other values were expressed relative to this value^24^). For paired-pulse measures, the conditioned (C) MEP was expressed as a ratio of the non-conditioned (NC) MEP (C/NC). To reduce the influence of outliers, we used an a priori trimmed mean procedure, in which the highest and lowest values of each set of 16 MEPs were discarded^51^. Any univariate outliers identified (z ± 3.29; four in the HD group and four in the Control group) were adjusted to a unit higher than the next most extreme score in their respective condition. Due to technical constraints, two values were not obtained at Baseline for a control participant in the Rest condition, and two values were not obtained at the Post-iTBS 15-min time-point for an HD participant in the Rest condition. These values were imputed using mean value replacement. Prior to analysis, a visual inspection of histograms showed positive skew for SICI and ICF variables. To satisfy the normality assumption for statistical analyses, we transformed these variables using a natural logarithm, however, untransformed data are presented in Figures.

Frequentist analyses were conducted using the Statistical Package for the Social Sciences (Version 25; Chicago, IL, USA), with alpha set to 0.05. To determine differences between groups in the absence of exercise, we compared the HD and control groups on TMS measures at baseline, by undertaking two-way mixed model analysis of variance (ANOVA), with Exercise Session as the within-subjects variable and Group as the between-subjects variable. Separate ANOVAs were conducted for each of the TMS measures of interest (Rest threshold, Active threshold, CME, SICI, ICF). Where appropriate, Greenhouse–Geisser epsilon was used to correct for violations of sphericity. To examine differences in synaptic plasticity following exercise across groups, we used three-way mixed model ANOVAs, where Exercise Session and Time were within-subjects factors, and Group was the between subjects factor. Separate ANOVAs were conducted for the TMS measures of CME, SICI and ICF. Significant main effects and interactions were examined further using one-way ANOVAs. We also performed planned group comparisons of change in these TMS measures across each session using *t* tests. To assess magnitude of effects, we calculated partial eta-squared (*η*_p_^2^)^52^.

Given our small sample size, and to more directly compare the effect of exercise condition on change in neuroplasticity measures (CME, SICI, ICF) post iTBS within each group, Bayesian analysis is also reported. Bayesian analyses are helpful in the context of non-significant results, as they provide quantification of evidence in favor of the null hypothesis, and therefore indicate whether the observed data truly provide evidence of absence, or simply indicate an absence of evidence^53,54^. In order to assess the strength of the evidence for the presence or absence of an effect of exercise on the neuroplasticity measures (CME, SICI and ICF) within each group separately, one-way repeated measures Bayesian ANOVAs were run for each group, where Exercise Session was the within-subjects factor, using JASP software^55^. Specifically, we calculated Bayes Factors corresponding to evidence in favor of H_1_ relative to the H_0_ (BF_10_), using default priors for ANOVA^56^. By convention, only Bayes factor values below 0.33 or above 3 are considered noteworthy, with a BF_10_ value ≥ 3 indicating that the alternative hypothesis is ≥ 3 times more likely than the null hypothesis, and conversely a BF_10_ value ≤ 0.33 indicating the null hypothesis is ≥ 3 times more likely than the alternative hypothesis, i.e., at least moderate evidence in favor of the alternative, or null hypothesis, respectively^57,58^. Values between 0.33 and 3 are considered inconclusive and only anecdotal evidence in favor of either hypothesis. ANOVA results with a Bayes Factors ≥ 3 were followed up with post hoc comparisons based on the default Bayesian *t* test with a Cauchy prior.

## Results

The results from the healthy control group have been reported before^26^, and are included here only to provide a comparison with the HD group.

### Baseline motor cortex neurophysiology

The HD and control groups were similar in terms of baseline resting and active motor cortex thresholds, and facilitation (ICF), with no significant main effects of Group or Session, and no interaction effects (Table 1 displays main effects of Group results for these variables). HD participants showed significantly less inhibition (SICI) at baseline than controls across all sessions, represented by a significant main effect of Group (F_1,32_ = 5.11, *p* = 0.03, η_p_^2^ = 0.14).

### Cortico-motor excitability

**Figure 2:**
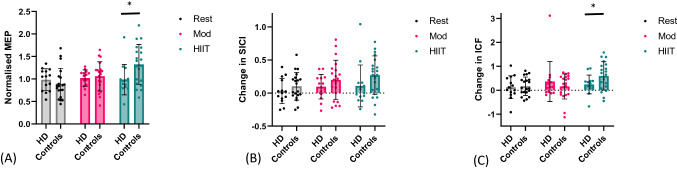
Effect of high-intensity interval training (HIIT), moderate-intensity continuous training (MICT) and rest on TMS measures for each group. Data represent the mean (standard deviation), with individual participant values also shown. *ITBS* intermittent theta burst stimulation. (**A**) Cortico-motor excitability (CME), average post-iTBS MEP, normalised from post-exercise time-point. (**B**) Short-interval intracortical inhibition (SICI). Change from pre-exercise baseline to average post-iTBS time-points. (**C**) Intracortical facilitation (ICF). Change from pre-exercise baseline to average post-iTBS time-points. *p < .05.

**Figure 3:**
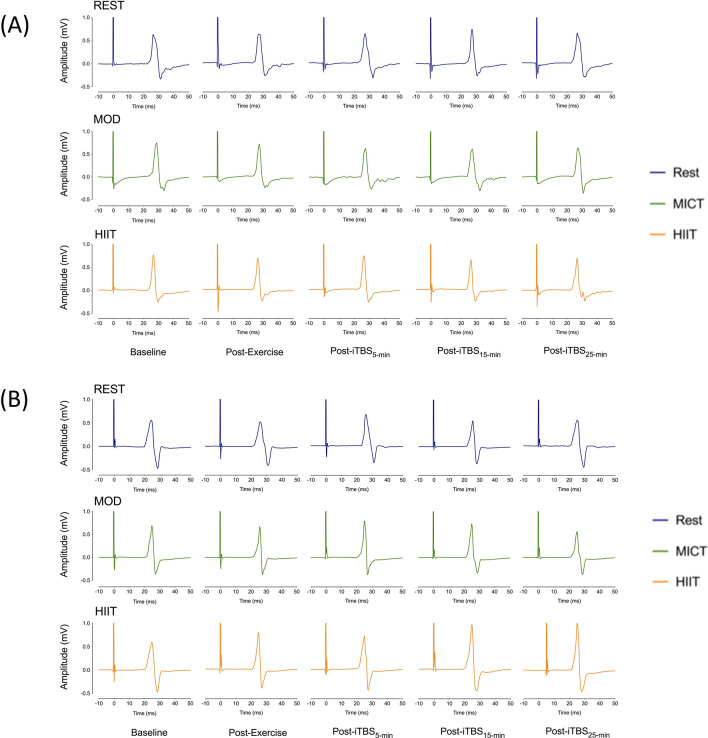
Examples of raw single motor-evoked potentials (MEPs) from one HD participant (**A**) and one control participant (**B**) during rest, moderate-intensity continuous training (MICT) and high-intensity interval training (HIIT). These MEPs were selected to illustrate the changes in cortico-motor excitability from baseline to post intermittent theta burst stimulation (iTBS) in the HIIT condition for control participants, but not HD participants.

We examined the effect of exercise on CME by normalizing the average post-iTBS MEP to the post-exercise time-point. Two-way ANOVA revealed a significant Group x Session interaction, (F_2,64_ = 3.72, *p* = 0.03, η_p_^2^ = 0.10), where the Control group showed significantly larger MEPs following iTBS in the high-intensity exercise condition than the HD group (*t* (32) = 2.39, *p* = 0.02), but there were no significant differences in MEPs between groups in either the Moderate intensity (*t* (32) = 0.41, *p* = 0.70) or Rest condition (*t* (32) = − 0.83, *p* = 0.42) (Fig. 2a). For the HD group, the Bayesian one-way repeated measures ANOVA provided moderate evidence in favor of the null hypothesis, that is, that neither high- nor moderate-intensity exercise boosted CME post-iTBS (B_10_ = 0.18; null hypothesis approximately 5.4 times more likely than the alterative hypothesis). In contrast, the control group showed very strong evidence of a significant difference between sessions, (BF_10_ = 36.23), with post-hoc comparisons revealing strong evidence that high intensity exercise increased CME post-iTBS compared to rest (BF_10_ = 20.15). In contrast, there was no convincing evidence that high-intensity exercise enhanced CME compared to moderate-intensity exercise (BF_10_ = 1.35), nor that moderate-intensity exercise increased CME in comparison to rest (BF_10_ = 0.68). Figure 3 shows examples of raw, single MEPs at each time-point within each condition from a representative HD participant and control participant, which demonstrate these patterns in CME seen in each group.

### Short-interval intracortical inhibition

A 3 Session × 5 Time × 2 Group mixed model ANOVA revealed a significant reduction in inhibition following high- and moderate-intensity exercise and iTBS in the control group but not the HD group, reflected by a significant Session × Group interaction effect (F_2,64_ = 3.45, *p* = 0.04, η_p_^2^ = 0.10), and a significant main effect of Time (F_4,128_ = 3.35, *p* = 0.01, η_p_^2^ = 0.10). There were no main effects of Session, or Group, or interaction effects between Session × Time, Time × Group, or Session × Time × Group. The change in SICI from the Pre-Exercise time-point to the average of the post-iTBS time-points for each group is shown in Fig. 2b. There were no significant differences between groups for any of the conditions (Rest: (*t* (32) = 1.05, *p* = 0.30); Moderate-intensity condition: (*t* (32) = 1.1, *p* = 0.27); High-intensity condition: (*t* (32) = 1.5, *p* = 0.14)). For the HD group, Bayesian one-way repeated-measures ANOVA provided moderate evidence in favor of the null hypothesis, that is, that neither high- nor moderate-intensity exercise reduced SICI post iTBS (BF_10_ = 0.27). For the control group, the evidence that the change in SICI differed between sessions was inconclusive (BF_10_ = 1.20).

### Intracortical facilitation

A 3 Session × 5 Time × 2 Group mixed model ANOVA revealed a significant increase in facilitation following high-intensity exercise and iTBS in the control group but not the HD group, reflected by a significant Session x Group interaction effect (F_2,64_ = 3.93, *p* = 0.03, η_p_^2^ = 0.11), and a significant main effect of Time (F_4,128_ = 5.83, *p* < 0.001, η_p_^2^ = 0.15). There were no main effects of Session, or Group, or interaction effects between Session × Time, Time × Group, or Session × Time × Group. The change in ICF from the Pre-Exercise time-point to the average of the post-iTBS time-points for each group is shown in Fig. 2c. There was significantly more facilitation following high-intensity exercise and iTBS in the control group in comparison to the HD group, (*t* (32) = 2.13, *p* = 0.04), but there were no between group differences in the moderate exercise (*t* (32) = − 0.89, *p* = 0.38) or rest (*t* (32) = 0.09, *p* = 0.93) conditions. For the HD group, Bayesian one-way ANOVA again revealed moderate evidence in favor of the null hypothesis, indicating neither the high- nor moderate-intensity exercise had any effect on ICF post iTBS (BF_10_ = 0.27). In contrast, for the control group, there was strong evidence in favor of the alternative hypothesis, that ICF differed across groups (BF_10_ = 14.39), with moderate evidence that high-intensity exercise induced a larger increase in ICF following iTBS than rest (BF_10_ = 6.20), but no conclusive evidence that high-intensity exercise induced a larger increase in ICF following iTBS than moderate-intensity exercise (BF10 = 2.38). In comparison, there was moderate evidence that moderate-intensity exercise had no effect on ICF, in comparison to rest (BF_10_ = 0.24).

### Relationships between demographic and clinical characteristics and TMS outcomes

We investigated relationships between age, self-reported physical activity levels, mood symptoms, and delta change values (Post-iTBS—Baseline) in the TMS measures (CME, SICI, or ICF), using Pearson correlations (Spearman correlations for non-normally distributed variables) and a more conservative significance level of *p* < 0.01 due to multiple comparisons. There were no significant relationships between these variables in either group across any of the three conditions. There were also no significant relationships between DBS and any of the TMS measures in the HD group.

## Discussion

Contrary to our first hypothesis, the premanifest and early HD participants did not show enhancement of synaptic plasticity following high-intensity interval exercise, in comparison to either moderate intensity exercise or rest. Specifically, the HD group showed no increases in cortico-motor excitability, glutamatergic facilitation, or decreases in GABA_A_ergic inhibition following either high- or moderate-intensity exercise, a finding which was further supported by the follow-up Bayesian analyses. The HD group showed lower inhibition at baseline which may have attenuated the effect of exercise on plasticity, consistent with our alternative hypothesis. Taken together, the current findings indicate that HD may be associated with an abnormal, attenuated plasticity response to an acute bout of cardiorespiratory exercise in premanifest and early HD, which has implications for the design of exercise interventions in this population.

Several possible mechanisms may account for the absence of exercise induced plasticity in the HD group. The first is that the HD group showed low baseline levels of GABA_A_ergic intracortical inhibition compared to controls, which has been observed in several previous studies^29,30,59^. In healthy adults, exercise transiently reduces GABA_A_ergic intracortical inhibition^17,18^. In HD, due to homeostatic mechanisms, the already reduced SICI in HD at baseline may have precluded further reductions in response to exercise^60^. However, baseline levels of corticomotor excitability and intracortical facilitation were not different than controls. Given the HD group also did not show the expected plasticity response for these variables, homeostatic mechanisms are unlikely to wholly account for the lack of responsivity of neuroplasticity to exercise. Instead, this attenuated synaptic plasticity response might also be attributable to altered dopamine signaling, or reduced production of BDNF, both of which have previously been demonstrated in HD^61,62^. In healthy adults, acute exercise triggers the release of lactate, dopamine, and the synthesis and release of BDNF^63^, which are associated with decreased cortical inhibition and corresponding increase in neuroplasticity^63,64^. Future research should investigate the effects of exercise on dopamine and BDNF release in people with the HD CAG-expansion, to better understand these potential underlying mechanisms.

An additional explanation for the lack of plasticity response in the HD group, is that exercise applied prior to the application of iTBS may have triggered a homeostatic inhibitory response^65^. However, we note that the healthy control group did not show evidence of this kind of homeostatic suppression of plasticity, and generally, exercise by itself does not induce changes in plasticity, but rather is thought to create an environment conducive for plasticity^16^. Further, recent research investigating homeostatic metaplasticity in HD mouse models reported disruption of these processes in the mouse brains^66^, although this has not been directly studied in humans. In the current study, the break between the cessation of exercise and the application of iTBS was approximately 15 min (10-min cool down followed by TMS measures). A previous study^67^ found that two trains of iTBS separated by 5 min resulted in an inhibitory effect of CME, whereas a 15 min interval resulted in a facilitatory effect on CME. The authors suggested that the 5-min interval may have triggered homeostatic plasticity, whereas the longer interval may have induced a more stable LTP-like effect. Therefore, even if exercise did induce LTP-like plasticity, the timings used in the current study mean that homeostatic plasticity is unlikely to account for the attenuated plasticity effect in seen in the HD group.

These findings have important implications for studies of exercise in HD, as attenuated brain responses to exercise may contribute to the mixed outcomes reported for exercise interventions in HD to date. Although some non-randomized controlled trials of exercise interventions have reported promising results, a recent meta-analysis of motor and cognitive effects from randomized controlled trials indicated no significant effects from the interventions on either the primary outcome (UHDRS motor score) or secondary outcomes (cognitive, health status or physical)^11^. The current findings indicate that more research is urgently needed to understand under what circumstances exercise may elicit an optimal neurophysiological response in HD (e.g., by investigating response to different types of exercise, or if there are effects over the longer-term following multiple exercise sessions), to inform the future direction of exercise intervention research in this population.

Our findings contrast with reports of exercise effects on the brain in HD mouse models. For example, wheel running in R6/1 transgenic HD mice increases BDNF gene expression^3,4^ and delays onset of motor signs^7^, whereas treadmill exercise in CAG140 knock-in HD mice restores dopamine D2 receptor expression^6^. The exercise interventions in these studies were long-term, however, rather than a single bout of exercise as implemented in the current study. In HD, longer-term exercise may be needed to have potent beneficial effects on brain chemistry, such as BDNF and dopamine levels.

Our study included people with the HD CAG expansion in very early stage, as much as 43 years before predicted onset, but also participants who had already been diagnosed. Manifest HD participants may have had too much neurodegeneration to respond optimally to exercise, although we did not find support for this possibility, in that disease burden score in our HD sample was not associated with the size of plasticity response following exercise. Given our small sample size, however, replication with a larger sample would be needed before any conclusions could be drawn regarding whether neuroplasticity alterations in HD track with disease progression. An additional consideration was the age of the HD participants, as some participants were older (3 participants were aged over 50 years), and in healthy populations, older participants have shown attenuated neuroplasticity response to TMS protocols^68^. In the current study, however, the control group did not show an attenuated plasticity response, despite some control participants also being older (4 participants aged over 50 years), making age a less likely explanation for the lower plasticity response seen in the HD group. Further, age was not significantly associated with the measures of plasticity in either group. In HD, there is a relationship between CAG expansion, age and the timing and severity of disease onset, whereby people with larger CAG expansions tend to have a younger and more severe onset of symptoms in HD^1,39^. Therefore, future research should investigate the relationships between age, disease severity and neuroplasticity response in larger HD samples.

Unlike the control group, who showed an increased facilitatory response to iTBS following high-intensity exercise, HD participants did not show any change to iTBS response following either exercise intensity. Only one previous study has utilized a theta-burst paradigm within a HD population. Orth et al.^28^ investigated responses to continuous TBS (cTBS) in a mixed premanifest and manifest HD group and found that cTBS had no effect on inhibition in HD, whereas cTBS resulted in significantly increased inhibition in the control group. Our current study extends this to show that in a premanifest and early HD sample, there was no detectable effect of iTBS on excitability, inhibition, or facilitation, either alone or primed with moderate- or high-intensity exercise. This suggests that these neuroplasticity mechanisms are affected early on in the disease course of HD, which has important implications for the consideration of non-invasive brain stimulation interventions in HD. Future research could also investigate whether exercise paired with a non-invasive brain stimulation protocol that suppresses excitability, such as continuous TBS^69^, might be more effective in altering plasticity in HD, given the lower baseline SICI seen in this population.

The current study has several limitations. We did not include an objective measure of cardiorespiratory fitness (e.g., VO_2max_), and therefore we could not examine any relationships between physical fitness and synaptic plasticity. However, groups did not differ on self-reported physical activity levels, resting heart rate, or objective measures of exercise performance, suggesting our groups were reasonably well matched. We also did not assess the potential role of BDNF genotype as a mediator of the plasticity response within the HD group, due to sample size limitations. Our group and others have previously found BDNF genotype to mediate the plasticity response to iTBS in healthy adults^26,70^. Further, our small sample size, particularly in the HD group, means that we may have been underpowered to detect an effect of exercise on neuroplasticity in the HD sample. Our follow up Bayesian analyses, however, provide moderate evidence that the results likely reflect a true absence of effect of exercise.

## Conclusions

In conclusion, the current study is the first to investigate the acute effects of exercise and iTBS on measures of motor cortex plasticity in premanifest and early HD. In contrast to the control group, we found no effect of either high- or moderate-intensity exercise and iTBS on the individual plasticity measures in the HD participants. These findings call into question the assumption of the benefits of exercise to the brain in HD, and demonstrate the need for future research to better understand exercise and plasticity mechanisms in people with the HD gene-expansion. Research is needed to investigate whether optimal neuroplasticity responses are elicited following longer cardiovascular exercise intervention protocols, such as those over days or weeks, as well as the effect of alternative exercise and non-invasive brain stimulation protocols. Answering these questions will be essential for creating physical activity guidelines and designing exercise interventions in HD that are most likely to have significant clinical benefit.
